# 100 telephone conversations about malignant hyperthermia

**DOI:** 10.1186/1471-2253-14-S1-P2

**Published:** 2014-08-18

**Authors:** Bodil Petersen, Udo X Kaisers, Henrik Rueffert

**Affiliations:** 1 Department of Anesthesiology and Intensive Care Medicine, University of Leipzig, Medical Faculty, Leipzig, 04103, Germany; 2 Department of Anaesthesiology and Intensive Care Medicine, Hospital Leipziger Land, Borna, 05542, Germany

## Background

We sought to analyse the telephone calls concerning MH to the Department of Anaesthesiology and Intensive Care Medicine, University Hospital, Leipzig, Germany.

## Material and methods

A total of 100 documented telephone inquiries from January 2011 until March 2014 were summarized, analysed for caller and cause of contact.

## Results

100 inquiries were analysed: 76 patients and 24 medical doctors called. Out of the 24 medical doctors, 16 were anaesthesiologists. Reason for malignant hyperthermia (MH) inquiry: Positive family anamneses (37%), own anaesthetic accident (29%) or general information about MH (34%).

In the group of positive family anamneses, 34 patients with MH or their relatives and three medical doctors sought information. The described anaesthetic accident ranged from reaction to death during an operative procedure. Inquiries based on an anaesthetic accident were addressed by a patient 20 times and by a medical doctor 9 times.

Some curious questions were asked:

Anaesthetist: “Patient had an anaesthetic incident forty years ago. What should I do?” (anaesthetic management)

Patient: “My anaesthetist refused the anaesthesia as long as I´m not tested for MH!” (suspicious family history)

Intensive care physician: “Our patient on the ICU must immediately be tested for MH because he has a rhabdomyolysis and we don´t no why!” (no triggering substances!)

Patient: “My uncle had problems during anaesthesia. Now our family should be tested. How do I proceed?”

General practitioner: “MH was suspected in a muscular biopsy.”

## Conclusions

MH is a known but with uncertainties connected disorder especially in association with muscular diseases. Approximately three-quarter of inquiries were made from patients. Patients profit from the care of specialized MH centre. However, further educational work about MH by specialized centres is still necessary.

